# Correlates of turnover intention among nursing staff in the COVID-19 pandemic: a systematic review

**DOI:** 10.1186/s12912-022-00949-4

**Published:** 2022-07-04

**Authors:** Katharina Herta Tolksdorf, Ulla Tischler, Katherina Heinrichs

**Affiliations:** 1 Charité – Universitätsmedizin Berlin, corporate member of Freie Universität Berlin and Humboldt-Universität zu Berlin, Berlin, Germany; 2 Institute of Health and Nursing Science, Charité – Universitätsmedizin Berlin, corporate member of Freie Universität Berlin and Humboldt-Universität zu Berlin, Augustenburger Platz 1, 13353 Berlin, Germany

**Keywords:** COVID-19, Nurses, Retention, Review, Turnover intention

## Abstract

**Background:**

During the COVID-19 pandemic, shortage of nursing staff became even more evident. Nurses experienced great strain, putting them at risk to leave their jobs. Individual and organizational factors were known to be associated with nurses’ turnover intention before the pandemic. The knowledge of factors associated with turnover intention during the pandemic could help to foster nurses’ retention. Therefore, this review aims to identify factors associated with nurses’ turnover intention during the COVID-19 pandemic.

**Methods:**

After a systematic search of six databases, the resulting records were screened in a two-step process based on pre-defined inclusion and exclusion criteria. The included quantitative studies were synthesized qualitatively due to their methodological heterogeneity.

**Results:**

A total of 19 articles were included in the analysis. Individual factors such as health factors or psychological symptoms and demographic characteristics were associated with nurses’ turnover intention. Organizational factors associated with turnover intention were e.g., caring for COVID-19 patients, low job control or high job demands, and moral distress. Resilience and supporting leadership could mitigate adverse associations with turnover intention.

**Conclusions:**

The results help to identify high-risk groups according to individual factors and to develop possible interventions, such as trainings for nurses and their superiors, addressing individual and organizational factors. Future research should focus on longitudinal designs applying carefully defined concepts of turnover intention.

**Supplementary Information:**

The online version contains supplementary material available at 10.1186/s12912-022-00949-4.

## Introduction

In March 2020, the World Health Organization (WHO) declared the Coronavirus Disease 2019 (COVID-19) a global pandemic [1]. Nurses, who make up 59% of the world’s health workforce, play a central role in maintaining patient care during this ongoing crisis. At the same time, there is a nursing shortage of nearly six million nurses worldwide today [2] which puts this professional group under great pressure [3]. Besides physical challenges and high workload, there are many cognitive and emotional challenges nurses have to deal with. For instance, many nurses were trained for new tasks or redeployed [4–7]. Additionally, they have to deal with severe disease progression among patients and are at risk to get infected themselves [8, 9]. These continuous strains are associated with the deterioration of the nurses’ mental health: symptoms of depression, anxiety, and inadequate sleeping hours were reported [10] as well as post-traumatic stress disorder (PTSD) among frontline nurses, who have direct contact with COVID-19 patients [11, 12].

Even prior to the COVID-19 pandemic, high job strain was associated with turnover intention among nurses [13–16]. Turnover intention is an important precursor of actual turnover behaviour [17], which is one of the main contributors to the nursing staff shortage [18]. During the COVID-19 pandemic, turnover rates among nurses increased [19, 20].

Although there are inconsistencies in definition, turnover intention may be understood as the desire of an employee to quit their current job within a certain time period [21]. Furthermore, Takase [22] described the construct of turnover intention as a multi-stage process, which starts with psychological responses to negative aspects of the current job and could lead to the decision to quit. The decision to leave the job could finally result in turnover behaviour. Although nurses leaving their profession entirely could be considered the major problem, turnover within the profession can also cause substantial costs, e.g., due to decreased productivity and training costs for new hires [23].

A systematic review of systematic reviews before the COVID-19 pandemic examined several factors which are associated with turnover intention [17]. In this review, factors were clustered into individual, job-related, interpersonal, and organizational factors. Individual factors positively associated with turnover intention were stress, burnout, and job dissatisfaction, whereas associations of turnover intention with age, gender, and educational level were inconsistent [17]. Within the domains of job-related, interpersonal, and organizational factors, the factors workload and certain shift patterns, satisfaction with supervision, and staff shortage were found to possibly influence nurses’ turnover intention, amongst others [17]. At this point, it should be noted that the formation of turnover intention is a complex process comprising several different factors [24], which might have changed with the onset of the COVID-19 pandemic. The knowledge of factors associated with turnover intention during the COVID-19 pandemic could help to foster nurses’ retention, especially in times of crises. Therefore, this review aims to identify factors associated with nurses’ turnover intention during the COVID-19 pandemic.

## Methods

### Search strategy

To achieve the study aim, a systematic literature search was conducted. Based on the three PEO components [25] population (i.e., nursing staff), exposure (i.e., COVID-19 pandemic), and outcome (i.e., turnover intention), a search string was built (see Additional file 1). As mentioned above, there is no clear definition of “turnover intention”. This study refers to all types: “Turnover intention” is used as an umbrella term for “intention to leave”, “intention to quit”, “consideration of attrition”, and “consideration of resignation” [21, 22]. Positively formulated terms such as “intention to stay” are excluded from the scope of this review because they describe different constructs with different work-related factors as correlates [26, 27].The search string included synonyms in English and German with truncation as well as Medical Subject Heading terms with automatic explosion and was applied to all fields. A filter for publication in the years 2020 and 2021 was set. The search was conducted on 14 October 2021. In order not to miss any new publications during the analysis and writing process, an alert for the search was installed. The last alert results were accounted for on 31 December 2021.

### Inclusion and exclusion criteria

The inclusion criteria were: a) nursing staff with patient contact as the target population, b) data assessment during the COVID-19 pandemic, and c) original peer-reviewed articles d) in German or English e) using a quantitative design with f) turnover intention as an outcome and g) at least one potential correlate, i.e., working conditions or demographic characteristics. Exclusion criteria were: a) target populations consisting of others than fully educated nursing staff (with patient contact) without profession-specific analyses and b) solely positive constructs, e.g., intention to stay, as outcomes. During data extraction, a third exclusion criterion was formulated: c) turnover intention assessment item implies possible correlates (e.g., “considered leaving nursing because of the workload, stress, and fear” [28]) other than COVID-19 (e.g., “Due to COVID-19, next year I will probably look for a new job outside this organization” [29]).

### Search and selection process

To cover a wide range of medical, psychological, and nursing science sources, the databases MEDLINE, CINAHL, PsycINFO, PSYNDEX, PsycArticles, and SocINDEX were searched via the platform EBSCOhost. The resulting articles were exported to the bibliographic management software Endnote 20.1. Duplicates were automatically eliminated by EBSCOhost comparing the citation metadata title, author, date published, and ISSN or journal name or ISBN. Two researchers (K.T. and K.H.) screened titles and abstracts independently using Rayyan, a web application to perform collaborative systematic reviews. Duplicates not detected by EBSCOhost were removed. Conflicting judgments from both reviewers were discussed until a consensus was reached. After this, full-text articles were screened based on the inclusion and exclusion criteria, again independently by the same researchers (K.T. and K.H.). Eventually, data were extracted and quality appraisal performed, evenly divided between all authors. K.H. checked all extracted data by comparing them with the original articles. According to Stone et al. [30], we did not exclude articles of low quality in order to avoid selection bias. For the included articles, the references were tracked and citing literature was cross-checked on 31 December 2021, again evenly divided between all authors. The final set of articles was synthesized qualitatively because of the methodological heterogeneity of the found studies.

## Results

**Fig. 1:**
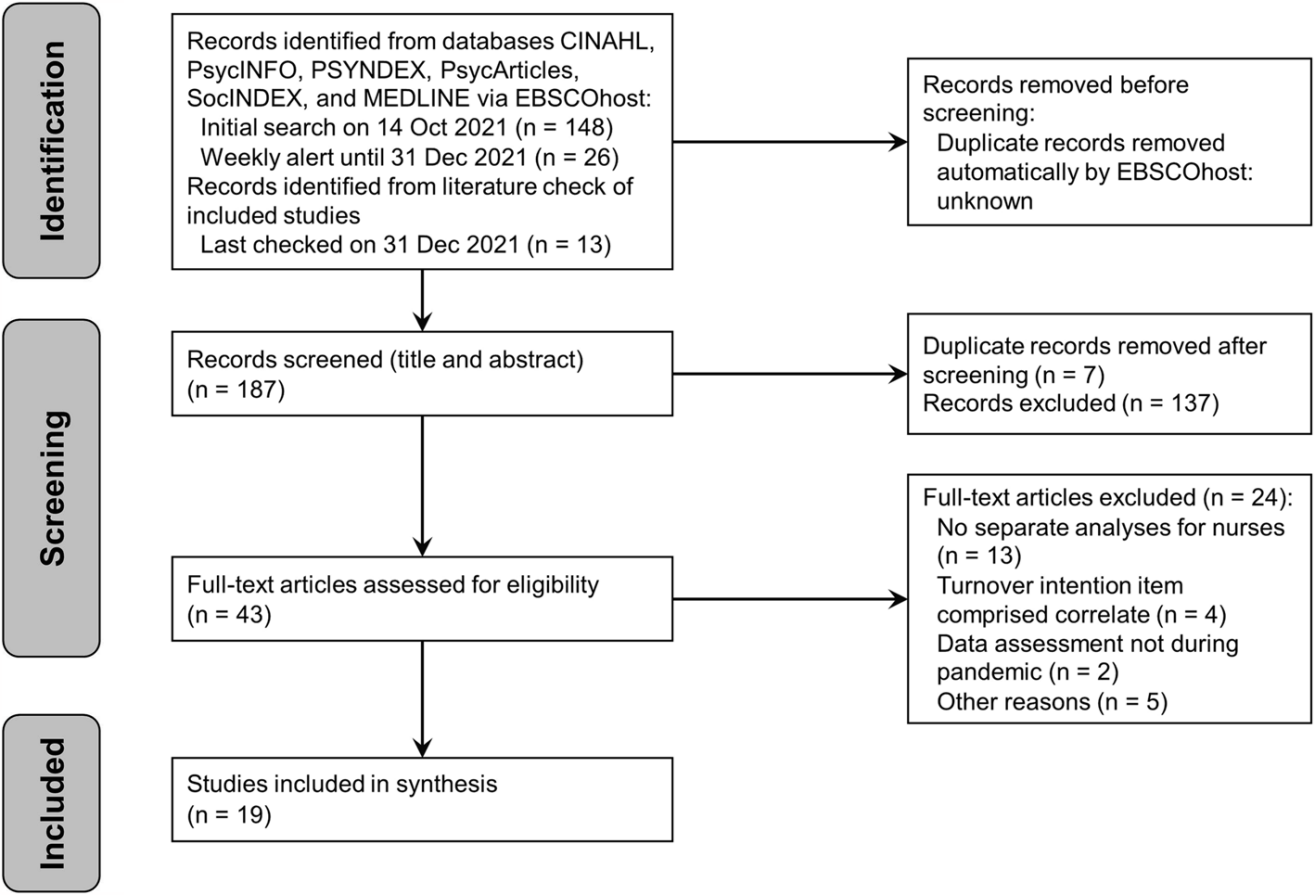
Flow Diagram of search and selection process

Figure 1 displays the selection process. The initial search resulted in 148 records, the ongoing alert in 26 further records. The cross-checking of literature delivered 13 further results. Out of the 187 articles which entered screening of titles and abstracts, 14 received conflicting judgments from the two reviewers involved in this phase (K.T. and K.H.), leading to a Cohen’s kappa of κ = 0.8 (substantial agreement). Eventually, 43 articles entered full text analysis, during which it became clear that a third exclusion criterion was needed. Turnover intention was measured with different items across the studies, and some already implied correlates (e.g., “considered leaving nursing because of the workload, stress, and fear” [28]. Those studies were excluded. Eventually, 19 publications entered qualitative synthesis.

### Description of studies

Table 1 presents the data extracted from the 19 included publications. The studies were conducted in the Middle East (incl. Pakistan; *n* = 7), Southeast Asia (*n* = 5), North America (*n* = 3), Europe (*n* = 2), Australia, and Taiwan (*n* = 1, each) in the years 2020 and 2021 and reported samples between *n* = 64 and *n* = 1,705 (mean = 398). The instruments to assess turnover intention mostly comprised one or two items (*n* = 11), up to 15 items, but were not always described in detail. All studies except for one which followed a mixed-methods approach used a cross-sectional design analysing self-report questionnaire data. Several inference-statistical methods were applied to check the found associations for significance, mostly regression analyses (*n* = 12). The quality appraisal of the publications resulted in sum scores between 4 and 11 out of 12 possible points (mean = 8.6) [31].

**Table 1:** Description of included articles (*n* = 19)

No	Authors Year (first publication) Country	Aim of the study	Study population; setting Sample size; response rate (%) Age in years: mean (SD) unless otherwise specified; % female gender	Design, method (time of data collection) Assessed variables Turnover intention: assessment tool (answer format)	Statistical method. Significant results concerning turnover intention	Quality appraisal Specialist Unit for Review Evidence [31]
1	Alameddine et al. 2021 Lebanon [32]	To investigate the resilience levels, job satisfaction, and turnover intention and to determine the association between resilience, job satisfaction level, intention-to-quit, and exposure to violence	All nurses at a major public hospital and COVID-19 referral centre *n* = 265; 86.0 75.8% ages 30–45 years; 64.9	Cross-sectional design, online self-report questionnaire (spring 2020) Resilience; job satisfaction; occupational violence One item: “In the coming 12 months, do you intend to quit?” (likeliness, 4-point Likert scale)	Multiple linear regression. Turnover intention was negatively associated with resilience, i.e., those who were more likely to intend to quit were less resilient (*B* = -4.35; *β* = -0.14; *p* = .020)	9/12
2	Cornish et al. 2021 Australia [33]	To investigate the intentions of Australian emergency nurses to remain in or leave emergency nursing after the first year of the SARS-CoV-2 (COVID-19) pandemic	Australian emergency nurses *n* = 398; N/A 31,9% aged 30–39 years; 86,9	Cross-sectional design, online self-report questionnaire (February 15th to March 28th 2021) Contact with COVID-19 patients; feeling connected to organization/colleagues/team; pride in being an emergency department nurse (among others) Intention to leave emergency nursing within five years (not reported)	χ2-test. Turnover intention was associated with (a) receiving COVID-19 patients (*p* = 0.016), (b) not feeling more connected to their emergency department nursing team (*p* = 0.03), the broader emergency department team (*p* = 0.008), and their organization (*p* = 0.03) since the onset of the pandemic, and (c) lower pride in being an emergency department nurse (*p* = 0.014)	7/12
3	De los Santos & Labrague 2021 Philippines [34]	To investigate the association between fear of COVID-19 and job satisfaction, job stress, and turnover intentions	Frontline nurses deployed in community settings under the Human Resource for Health initiative *n* = 385; 96 32.7 (7.73); 84	Cross-sectional design, online self-report questionnaire (mid of June 2020) Fear of COVID-19; job satisfaction; job stress Organizational turnover intention: “Given the current situation, I am thinking about leaving this healthcare facility.” (5-point Likert scale); professional turnover intention: “Given the current situation, I am thinking of leaving nursing as a profession.” (agreement; 5-point Likert scale)	Multiple linear regression. An increased level of fear of COVID-19 was associated with increased organizational (*B* = 0.05; *β* = 0.24; *p* = .001) and professional (*B* = 0.05; *β* = 0.23; *p* = .001) turnover intentions	10/12
4	Elhanafy & El Hessewi 2021 Egypt [35]	To investigate the association between fear of COVID-19 and work satisfaction and turnover intentions	All nurses at inpatient units and critical care units of Damanhour National Medical Institute with at least 1-year experience in this work position *n* = 210; not reported 36.54 (8.91); 68.1	Cross-sectional design, self-report questionnaire (January to February 2021) Fear of COVID-19; work satisfaction Two single-item measures (O'Driscoll & Beehr, 1994): organizational and professional turnover intention (agreement; 5-point Likert scale)	Multiple linear regression. An increased level of fear of COVID-19 was associated with increased organizational (*B* = 0.428; *β* = 0.298; *p* = 0.001) and professional (*B* = 0.314; *β* = 0.219; *p* = 0.001) turnover intentions	9/12
5	Irshad et al. 2020 Pakistan [29]	To investigate outcomes, the underlying mechanism and boundary condition of the perceived threat of COVID-19	Nursing staff caring for COVID-19 patients with a minimum of six months of work experience n = 117; N/A 52,1% aged 21–30 years; 62.4	Cross-sectional design, online self-report questionnaire (not reported) Perceived threat of coronavirus; anxiety; ideological contract Three-item scale: (1) “Due to the current situation, I often think about quitting”; (2) “Lately, I have taken an interest in job offers in the newspaper due to COVID-19”; (3) “Due to COVID-19, next year I will probably look for a new job outside this organization” (agreement; 5-point Likert scale)	Pearson's correlation. Turnover intention was positively associated with (a) the perceived threat of COVID-19 (*r* = 0.47; *p* < .001) and (b) anxiety (*r* = 0.43; *p* < 0.001) and negatively associated with (c) ideological contract (*r* = -0.47; *p* < .001) Anxiety enhanced the relationship between perceived threat of COVID-19 and turnover intention. The combined effect of anxiety and ideological contract on turnover intention was negative and significant	7/12
6	Khattak et al. 2021 Pakistan [36]	To investigate the association between fear of COVID-19 and turnover intention, secondary trauma, and psychological distress and to investigate leadership support as a possible moderating variable	Nurses with or without direct contact with COVID-19 patients from 10 large hospitals of the Khyber Pakhtunkhwa province n = 380; 54.28 31.5 (not reported); 84.21	Cross-sectional design, not reported (not reported) Fear of COVID-19; psychological distress; secondary trauma; leadership support Two-item scale [37], sample item: “Given the current situation, I am thinking about leaving nursing as a profession” (not reported)	Regression analysis. Turnover intention was associated with fear of COVID-19 (*β* = 0.79; *p* < .05) Leadership support buffered this association	7/12
7	Labrague & De los Santos. 2021 a Philippines [38]	To investigate the association between fear of COVID-19 and psychological distress, work satisfaction as well as organizational and professional turnover intentions	Frontline nurses in one of five hospitals in the Philippines (three public hospitals and two private hospitals) *n* = 261; 87 30.95 (6.14); 73.6	Cross-sectional design, not reported (not reported) Fear of COVID-19; job satisfaction; job stress Two single-item measures [39] (organizational turnover intention: “Given the current situation, I am thinking about leaving this healthcare facility”; professional turnover intention: “Given the current situation, I am thinking of leaving nursing as a profession” (agreement; 5-point Likert scale)	Pearson's correlation. An increased level of fear of COVID-19 was associated with increased organizational (*r* = .295; *p* = .001) and professional (*r* = .188; *p* = .001) turnover intentions Multiple linear regression. An increased level of fear of COVID-19 was associated with increased organizational (*B* = .428; *β* = .298; *p* = .001) and professional (*B* = .314; *β* = .219; *p* = .001) turnover intentions COVID-19-related training (*p* = .022) lowered fear of COVID-19, with nurses who reported no COVID-19-related training and held part-time job experiencing higher levels of fear of COVID-19	8/12
8	Labrague & De los Santos. 2021b Philippines [40]	To investigate the association between compassion fatigue and job satisfaction, turnover intention, and care quality and to investigate resilience as a possible moderating variable	Frontline nurses working in selected hospitals in the previous six months and directly caring for suspected or infected COVID-19 patients *n* = 270; 90 34.86 (8.83); 74.5	Cross-sectional study, online self-report questionnaire (November 1 – December 1, 2020) Compassion fatigue; resilience; job satisfaction; quality of care One item: “Given the current situation, I am more likely to leave my profession” (agreement; 5-point Likert scale)	Pearson's correlation. Turnover intention was positively associated with compassion fatigue (*r* = .301; *p* = 0.001) and negatively associated with quality of care (*r* = -.128; p = 0.01) Multiple linear regression. Turnover intention was positively associated with compassion fatigue (*B* = .328; *β* = .301; *p* = 0.001) and negatively associated with resilience (*B* = -.336; *β* = .178; *p* = 0.004) Resilience partially mediated the association between compassion fatigue and turnover intention (*p* = 0.001)	10/12
9	Labrague et al. 2021 Philippines [41]	To investigate the association between nurses’ perceptions of COVID-19-associated discrimination and their resilience, mental health, and professional-turnover intention	Frontline nurses with at least six months of work experience in their current unit and directly caring for COVID-19 patients *n* = 259; 86 34.86 (8.83); 74.5	Cross-sectional study, online self-report questionnaire (November 1 – December 1, 2020) COVID-19-associated discrimination; resilience; mental health One item: “Given the current situation, I am more likely to leave my profession” (agreement; 5-point Likert scale)	Pearson's correlation. Turnover intention was positively associated with COVID-19-associated discrimination (*r* = .123; *p* < 0.01) and negatively associated with resilience (*r* = -.178; *p* < 0.001) Multiple linear regression. Turnover intention was positively associated with COVID-19-associated discrimination (*B* = .130; β = .123; *p* = 0.048) and negatively associated with resilience (*B* = -.336; *β* = -0.182, *p* = 0.004) Resilience mediated the association between COVID-19-associated discrimination and turnover intention (*p* = 0.107)	10/12
10	Lavoie-Tremblay. (2021) Canada [42]	To identify correlates of turnover intention related to the COVID-19 pandemic	Frontline nurses and practical nurses in Quebec *n* = 1705 (782 caring for COVID-19 patients); 11 41.10 (10.82); 87	Cross-sectional study, online self-report questionnaire (July 22 to November 16 2020) Job demands: perception of being in control vs. overwhelmed, self-infection, infection of a team member, provided care for COVID-19 patients; job resources: perception of preparedness, transformational leadership; strain and performance: chronic fatigue, work satisfaction, perceived quality of care Two single-item measures [39]: organizational (intention to leave their current work setting) and professional turnover intention (intention to leave the profession) (agreement; 7-point Likert scale)	*F*-test. Organizational turnover intention was associated with providing care to COVID-19 patients10 (*F* = 10.49; *p* < .01) t-test. Organizational and professional turnover intentions were positively associated with being poorly prepared (*t* = 11.16, *t* = 9.69, respectively; *p* < 0.001) and negatively associated with feeling in control of the situation at work (*t* = -13.19, *t* = -9.59, respectively; *p* < 0.001). Organizational turnover intention was negatively associated with self-infection or team infected answer “yes” (*t* = -3.01; *p* < 0.01) Regression model. Organizational turnover intention was positively associated with (a) chronic fatigue (*B* = .02; *β* = .31; p < .001) and negatively associated with (b) more experience in the setting (*B* = -.02; β = -.08; *p* < .001), (c) feeling in control of the situation at work (*B* = -.12; β = -.06, *p* < .01), (d) having a leader with a transformational leadership style (*B* = -.05; β = -.16; *p* < .001), and (e) work satisfaction (*B* = -.30; β = -.22; *p* < .001). Professional turnover was positively associated with (a) chronic fatigue (*B* = .03; β = .37; *p* < .001) and (b) caring for COVID-19 patients (*B* = .28; *β* = .07; *p* < .001) as well as negatively associated with (c) preparedness (*B* = -.17; *β* = -.07; *p* < .01), (d) having a leader with a transformational leadership style (*B* = -.02; *β* = -.08; *p* < .01), and (e) work satisfaction (*B* = -.19; β = -.15; *p* < .001)	11/12
11	Li et al. (2021) Taiwan [43]	To investigate the association between pandemic-related work conditions and adverse mental health among nurses and to investigate organizational strategies as possible moderating variables	Nurses working in medical facilities during the study period in Taiwan aged over 20 years *n* = 1499; N/A 36.2 (9.4); 96	Cross-sectional study, online self-report questionnaire (July to December 2020) Stressors: increasing working hours, caring for COVID-19 patients, occupational stigma, redeployment; organizational strategies: adequate protection equipment, adequate infection control measures, adequate education and training concerning COVID-19, compensation, support by the employer; burnout; depressive symptoms Intention to leave the current job during the pandemic in comparison with that before the pandemic (agreement; 5-point Likert scale)	Multivariate logistic regression. Turnover intention was positively associated with (a) redeployment (OR = 1.54; 95% CI: 1.13–2.10), (b) increased working hours (OR = 1.55; 95% CI: 1.16–2.07) and (c) occupational stigma (OR = 1.60; 95% CI: 1.22–2.10) as well as negatively associated with (d) education/training (OR = 0.61; 95% CI: 0.45–0.84) and (e) support by the employer (OR = 0,54; 95% CI: 0.36–0.81)	11/12
12	Lou et al. 2021 Canada [44]	To investigate coping strategies as possible moderating variables on the negative impact of stress and intentions to quit	Nurses and physicians of a university-affiliated tertiary care hospital network in Montreal *n* = 64; N/A 38,07 (11.14); 90,6	Cross-sectional study, self-report questionnaire (July 31 – August 15 2020) Depression; anxiety; stress; burnout; stressors; perceived Impact of stress on work; coping strategies Two items whether they were thinking about leaving their health care facility and their profession (Yes/No)	Logistic regression. Turnover intention was associated with burnout (*b* = 1.00; *SE* = 0.34; *p* = .003)	10/12
13	Mirzaei et al. 2021 Iran [45]	To investigate the association between turnover intention and psychosocial factors during the COVID-19 pandemic	Frontline nurses with more than six months of clinical work experience in Ardabil *n* = 479; 62 33.43 (6.77); 61.6	Cross-sectional study, online self-report questionnaire (June 2020) General health; job content; impact of event (COVID-19 pandemic) Turnover Intention Questionnaire [46]: 15 items (agreement; 5-point Likert scale)	Pearson's correlation. Turnover intention was positively associated with (a) post-traumatic stress disorder (*r* = .35), (b) general health (*r* = .31), (c) job demand (*r* = .30), (d) job insecurity (*r* = .27), and (e) job strain (*r* = .27; *p* ≤ .01 for all) and negatively associated with (f) social support (*r* = -.35; *p* < .01). Multiple linear regression: Turnover intention was positively associated with (a) job strain (*B* = 41.425; *β* = .624; *p* = .013), (b) decision latitude (*B* = .677; *β* = .445; *p* = .011), (c) post-traumatic stress disorder (*B* = .177; β = .238; *p* < .001), (d) general health (*B* = .582; *β* = .209; *p* < 0.001), and (e) married status (*B* = 2.976; *β* = .111; *p* = .009) as well as negatively associated with (f) female gender (*B* = -3.263; *β* = -.131; *p* = .002), (g) social support (*B* = -.714; *β* = -.243; *p* < .001) and (h) work position (*B* = -1.797; *β* = -.084; *p* = .038)	7/12
14	Nashwan et al. 2021 Qatar [47]	To identify correlates of turnover intention before and during COVID-19	Nurses working for Hamad Medical Corporation *n* = 512; 4.3 36.54 (7.42); 67.6	Cross-sectional study, online self-report questionnaire (August to September 2020) Stress level before and during COVID-19 Turnover Intention Scale (TIS-6): six items [48]	Wilcoxon signed-ranks test. Turnover intention was associated with (a) being single (*p* = 0.007), (b) 5–10 years of experience (vs. < 5 years; *p* = .023), (c) critical care deployment (vs. other areas; *p* = .047), 3–6 months of deployment (vs. > 6 months and < 2 months; *p* = 0.047), and (d) stress level (*p* < .001)	8/12
15	Özkan Şat et al. 2021 Turkey [49]	To investigate the association between nurses' exposure to violence and their professional commitment during the COVID-19 pandemic	Nurses working in public, private, and university health institutions *n* = 263; N/A 31.26 (7.17); 88.2	Cross-sectional study, online self-report questionnaire (October to December 2020) Status of exposure to violence during the COVID-19 pandemic process; professional commitment Professional turnover intention (not reported)	χ2-test. Turnover intention was associated with (a) exposure to physical violence (*p* = .011), (b) exposure to verbal-emotional-psychological violence (*p* = .002), (c) exposure to mobbing (*p* < .001), (d) department change (*p* = .043), (e) increased working hours (*p* < .001), and (f) increased number of patients (*p* = .004)	9/12
16	Petrișor et al. 2021 Romania [50]	To investigate the association between moral distress, depression, and anxiety, and turnover intention	Nurses in intensive care unit of the Emergency County Hospital Cluj-Napoca *n* = 79; N/A 37.05 (8.77); 89.87	Cross-sectional study, pen-and-paper self-report questionnaire (October 2020 to February 2021) Moral distress, anxiety, depression, number of years spent working in intensive care unit Present intention to leave (Yes/No)	t-test. Turnover intention was associated with higher moral distress, but only system-related factors (vs. patient- or team-related factors) differentiated between nurses intending to leave or not during the COVID-19 pandemic (*p* = 0.042)	9/12
17	Sheppard et al. 2021 USA [51]	To investigate the association between moral distress and turnover intention	All nurses at Sentara Williamsburg Regional Medical Center, Williamsburg, Virginia *n* = 129; 35,9 44.2 (12.8); 90.7	Cross-sectional study, online self-report questionnaire (July 2020 and August 2020) Moral distress One item: intent-to-leave question (Yes/No)	t-test. Turnover intention was associated with higher moral distress (*p* < .001) Stepwise binary logistic regression. Turnover intention was associated perceived issues with the work environment (OR = 9.09; 95% CI: 3.13–26.39) in third and final step	9/12
18	Widodo et al. 2021 Indonesia [52]	To investigate the association between organizational culture, pay satisfaction, and job satisfaction and intention to leave	Nurses at a private Hospital in Bantul *n* = 160; N/A not reported	Cross-sectional study, questionnaire (not reported) Organizational culture, pay satisfaction, job satisfaction Intention to leave (not reported)	t-test. Turnover intention was negatively associated with organizational culture, e.g., involvement and adaptability (*p* = 0.002) and nurses’ pay satisfaction (*p* = 0.019)	4/12
19	Wood et al. 2021 UK [53]	To investigate the experiences of advanced practice nurses during the COVID-19 pandemic, particularly in relation to safety, shortages, and retention	Advance practice nurses across primary and secondary care in all four nations of the UK *n* = 124; 51 41.94% aged ≥ 50 years; 86	Mixed-methods-design (two weeks in June 2020) Preparedness of the organization (impact on patient and staff safety, shortages of staff and equipment, concerns, ability to access guidelines and advice, policy regarding staff sickness) Intention to leave the organization or the profession (not reported)	Spearman’s rho test. Turnover intention among nurses was associated with (a) not being able to provide the same standard of care as they did before the crisis (*p* = 0.015), (b) not feeling their safety was prioritised (*p* = 0.01), and (c) not feeling there was sufficient communication from management about coronavirus planning (*p* = 0.03)	8/12

**Fig. 2:**
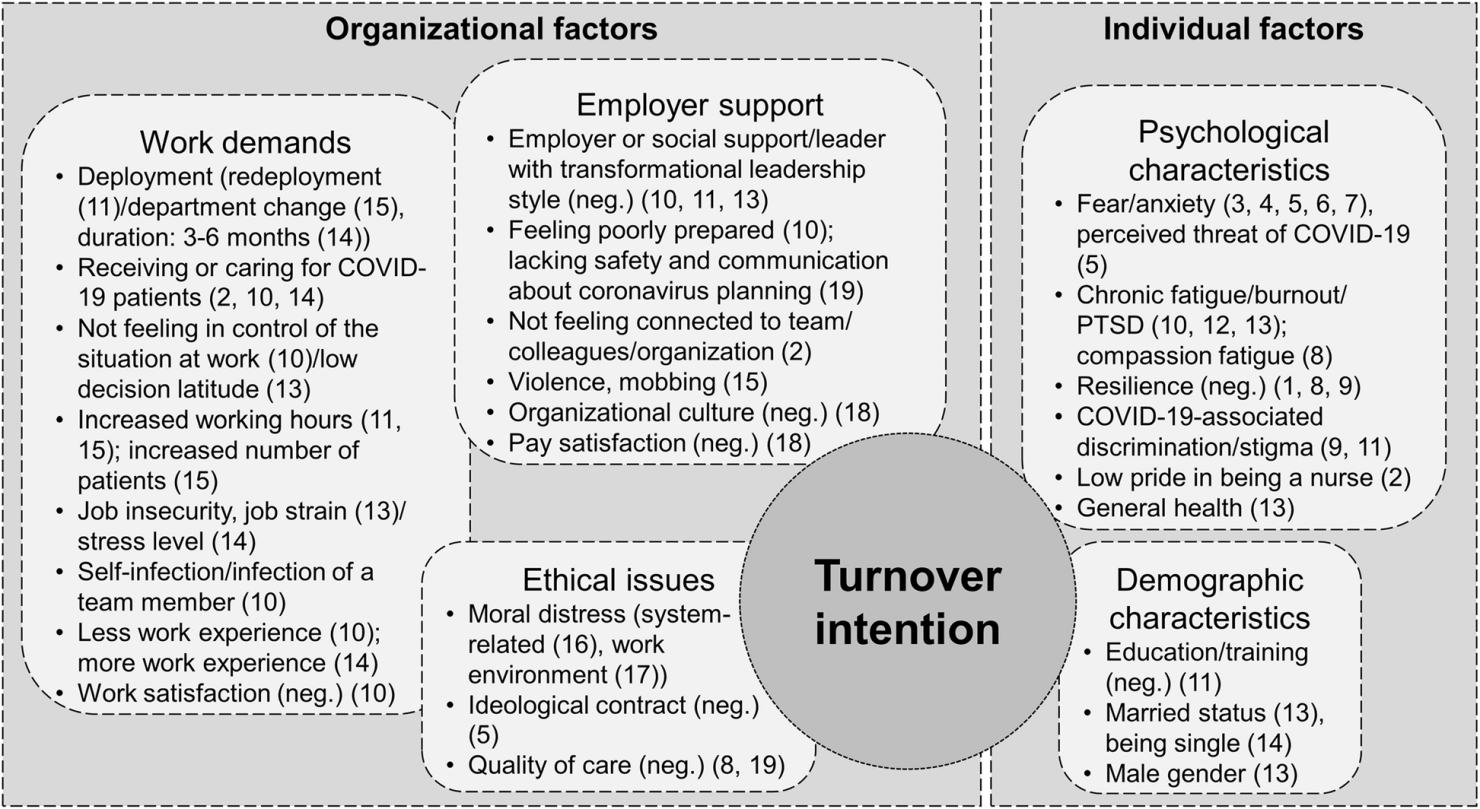
Factors associated with nurses’ turnover intention; legend: Numbers in parentheses refer to numbers of studies in Table 1

Figure 2 displays which factors showed significant associations with turnover intention. These factors can be clustered into the domains “individual factors” and “organizational factors”. Individual factors refer to psychological and demographic characteristics. Organizational factors comprise work demands, ethical issues, and aspects of employer support.

### Individual factors associated with turnover intention

During the COVID-19 pandemic, several psychological characteristics of the participating nurses were found to be associated with turnover intention (see Fig. 2). Several studies identified anxiety or fear of COVID-19 or perceived threat by COVID-19 as a correlate of turnover intention [29, 34–36, 38]. Besides general health [45], further psychological symptoms showed relationships with turnover intention, e.g., (compassion) fatigue, burnout, or PTSD symptoms [40, 42, 44, 45]. Discrimination [41], stigma [43], or low pride in being a nurse [33] were found to be associated with turnover intention, whereas resilience was negatively correlated with the outcome [32, 40, 41]. Male nurses reported lower levels of turnover intention [45], as did well-educated and specially trained nurses [43]. Regarding legal status, the results were inconclusive [45, 47].

### Organizational factors associated with turnover intention

Nurses who were (recently) deployed into critical care [43, 47, 49], received or cared for COVID-19 patients [33, 42, 47] or experienced an infection themselves or in their team reported higher levels of turnover intention [42]. Further job-related factors associated with turnover intention were low job control [42, 45], increased working hours or workload [43, 49], and job stress/strain in general as well as job insecurity [45, 47]. Nurses satisfied with their work or pay reported lower levels of turnover intention [42]. The findings concerning work experience were inconclusive [42, 47].

Several studies showed that ethical issues were associated with turnover intention, among them moral distress (positively) [50] as well as ideological contract [29] and quality of care (both negatively) [40, 53]. Employer support forms a third domain of organizational issues that are connected to turnover intention. Nurses who experienced supporting leadership, employer or social support, or a good organizational culture reported lower levels of turnover intention [42, 43, 45, 52]. When nurses felt poorly prepared [42] or not connected to their work environment [33], they reported higher levels of turnover intention. Lacking communication about coronavirus planning or lacking safety [53] as well as violence and mobbing [49] were additionally identified as correlates of turnover intention.

## Discussion

A wide range of correlates of nurses’ turnover intention during the COVID-19 pandemic could be found and roughly categorized in individual and organizational factors. They indicate detrimental work contexts and deteriorated working conditions during the pandemic. Furthermore, numerous psychological and sociodemographic characteristics appear to be pivotal regarding nurses’ turnover intention. In this context, it should be considered that factors may interact, reinforce or reduce each other's effects. For instance, low job control may result in high job strain (both organizational factors) and thus, stress-induced burnout (psychological, i.e., individual factor). Therefore, it must be assumed that several of the described factors are interdependent. Nevertheless, these factors open up opportunities for preventive interventions and show which nurses could particularly benefit from these.

### Individual factors

Psychological characteristics like anxiety, fear, and perceived threat of COVID-19 are factors associated with nurses’ turnover intention [29, 34–36, 38] which emphasizes the existential threat many nurses are experiencing in this pandemic. Also in the severe acute respiratory syndrome (SARS) pandemic, the perceived risk of death from SARS was an important predictor of nurses' turnover intention [54]. Leadership support [36] and ideological contract [29] may reduce the influence of fear on turnover intention. Even outside of crisis situations, the protective effect of ideological motives on turnover intention is known [55].

Turnover intention is also associated with (psychological) health factors and symptoms [40, 42, 44, 45]. The association between PTSD and turnover intention was also evident in the Middle East respiratory syndrome (MERS) epidemic, although, as with anxiety, leadership support was able to mitigate the relationship between PTSD symptoms and turnover intention [56]. Relieving health-impaired nursing staff through suited offers of health promotion interventions should be carefully considered, but more research is needed on how adaptive strategies can reduce the long-term impact of mental health threats like burnout [44]. Furthermore, personnel with leadership tasks should be trained to offer their staff the support they need especially in times of crises. The positive relationship between resilience and retention [32, 40, 41] has been consistently reported in pre-pandemic literature [57]. This review shows that resilience also mitigates the associations of compassion fatigue [40] and COVID-19-associated discrimination [41] with turnover intention. COVID-19 associated discrimination and stigma against nurses [41, 43] may be due to fear of infection [58] and were repeatedly described as a serious problem [59–61]. Strategies to improve retention could start with resilience promotion in the form of mindfulness-based stress therapy [62]. Firstly, since it is conceivable that the experience of stigmatization was particularly severe in the first phase of the pandemic due to the novelty and unfamiliarity of the virus [63], nurses’ resilience should be given special protection and fostering especially in the onset of a pandemic. Secondly, since a higher rate of compassion fatigue was mostly observed in nurses assigned to critical care units, emergency departments, and units designated for treating and managing patients with COVID-19 [64], nurses in these areas of work should be favoured for interventions. Additionally, public campaigns are conceivable to reduce nurses’ stigmatization and discrimination by the public and foster nurses’ pride in their work.

Concerning demographic characteristics, there are diverging results with regard to marital status and gender [45, 47]. This inconsistency is also reflected in the results of other studies. Depending on the population and setting examined, these play no [65] up to a significant role [66] in the context of nurses’ turnover intention. Analogous to the result of Mirzaei et al. [45], male nurses are more likely to be prone to turnover intention in some countries outside of pandemic situations [24] with strong differences in relevance of gender as a contributing factor to turnover intention between countries [67]. The diverging results regarding demographic factors can be due to different cultural conditions, family structures, and gender roles of nurses in different countries and settings. Age does not seem to be an associated factor of nurses’ turnover intention in the COVID-19 pandemic which is consistent with findings from the SARS pandemic in the years 2002 to 2004 [54].

### Organizational factors

High levels of social support from supervisors and colleagues seem to be associated with lower turnover intention [36, 43, 45]. This finding could be explained through the process of stress reduction [68] and has been evident under normal conditions [69] and in the MERS epidemic [56]. High leadership support may also be able to decrease the relationship between fear of COVID-19 and turnover intention [36]. The central role that supervisor support plays in influencing turnover intention has been widely acknowledged [17], and the positive effect of both leadership support [36, 43, 45] and leadership style [42] emphasizes the importance of social relationships, appreciation, and protection apart from purely monetary remuneration and also opens up opportunities for managers to improve satisfaction and retention among their employees. Nevertheless, pay satisfaction could also be a relevant factor [52] that should be considered in nurse retention efforts, but due to the poor quality of Widodo’s study [52] and the stronger evidence regarding the importance of leadership support in this review, the latter should be treated a priority.

Since factors related to organizational culture [52] and feelings towards team climate [33] as well as exposure to violence and mobbing [49] also seem to be relevant factors to nurses’ turnover intention, employers should not neglect interpersonal conditions in their organization and promote a positive workplace culture [70] as well as violence prevention measures and the implementation of support systems. Since it was also found that there was a correlation between turnover intention and nurses’ feeling that they are poorly prepared [42], their safety was not prioritized [53], and management communication on pandemic planning [53] was insufficient, those measures could create a well-founded sense that the safety of nurses is a high priority.

Working in [33, 42, 47] and redeployment to [43] COVID-19 patient care as well as a general department change [49] emerged as correlates of turnover intention. This work context seems to be particularly critical, which may be due to the numerous stress factors present in this setting. In the MERS epidemic, nurses involved in the direct care of suspected patients were also prone to increased turnover intention [49]. However, the SARS pandemic also showed that nurses caring for SARS patients were less likely to consider leaving. This finding was probably related to the nurses having received relevant training, which enabled them to better assess the risks so that they were less affected by fear [54]. The results of Li et al. could confirm this assumption, as they identified education and training concerning the COVID-19 pandemic as a protective factor for turnover intention [43]. This finding is in line with results from the SARS pandemic [71]. COVID-19 training should include information about the proper utilization of available resources, the nature of the virus, precautionary measures to avoid transmission, number of new and recovered cases reported per day as well as hospital protocols [36] and could be executed remotely to maintain social distancing. The results by Nashwan et al. [47] also point out that nurses dealing with COVID-19 patients in intensive care units for the past three to six months are particularly at risk of turnover intention. Even before the COVID-19 pandemic, intensive care nurses were known to be more likely to quit due to prolonged exposure to traumatic experiences and stress [72]. However, this component opens up a time window for targeted preventive interventions in the first three months of employment in COVID-19 intensive care or indicates that employment in this area could be limited in time. In addition, the NEXT study showed that there is also a six-month window between the formation of turnover intention and the actual dismissal of nurses during which preventive measures could be taken [73].

Increased working hours were found to be correlates of turnover intention in two studies [43, 49] as well as an increased number of patients [49] and higher job strain [45]. Additionally, nurses’ feelings of not being in control of the situation at work [42] and a low decision latitude and job insecurity [45] contribute to this intention. A high workload with little room for decision-making results in stress [74], which itself has the potential to contribute to turnover intention as well [44, 47]. However, the influence of workload on stress can again be reduced by supervisor support [75]. While the workload is unlikely to be reduced in the short term, especially in the early phase of a pandemic, in terms of decision latitude, it is possible to provide nurses with learning opportunities and participation in decision-making processes, e.g., concerning the implementation of infection protection measures. In addition, since the feeling that management communication on pandemic planning was insufficient [53] is associated with nurses’ turnover intention, involving nurses in the pandemic planning could at the same time eliminate this lack of communication. Furthermore, this could lead to a better understanding of the pandemic situation, reduce fear, and promote self-efficacy as well as job control. Stress reduction measures and learning adaptive coping strategies [44] could also reduce nurses’ turnover intention in this pandemic, if stress reduction itself is not feasible due to the crisis situation. Conversely, nurses with high levels of stress and maladaptive coping strategies can be identified as a particularly vulnerable group for turnover intention. Other studies have shown that stress and management problems outside of pandemics have both direct and indirect effects on job satisfaction and the intention to leave the company [76]. There is also a vicious circle between job stress and job satisfaction: intense stress leads to job dissatisfaction, which in turn increases the stress [77]. In the SARS pandemic, stress even proved to be the most important predictor of nurses' intention to leave [54]. Lavoie-Tremblay et al. [42] found that work satisfaction also could be a relevant protective factor of nurses’ turnover intention in the COVID-19 pandemic.

Differing results were present regarding work experience. Lavoie-Tremblay et al. [42] found that less experienced nurses are more prone to turnover intention while Nashwan et al. [47] found that nurses with five to ten years of work experience are more at risk. So far, both under normal circumstances [78, 79] and during the outbreaks of SARS [54] and MERS [56], less experienced nurses were more at risk of turnover intention. This finding could be explained by the fact that more experienced and therefore mostly older nurses find it more difficult to change jobs due to family obligations or a stronger sense of duty towards their organization due to longer employment [54]. The fact that Nashwan et al. [47] found experienced nurses more likely to report turnover intention may be due to a perceived threat to their own health or to the health of their families and may indicate the risk of losing experienced nurses in the current pandemic. However, because of the weaker quality of Nashwan’s study [47] and the fact that their results do not coincide with previous knowledge, this evidence should be considered with caution. Nevertheless, a self-infection with COVID-19 or that of a team member is likely to increase turnover intention [42] which emphasizes the need for sufficient and adequate personal protection equipment.

The moral distress nurses face due to system-related factors [50] and their work environment [51] could increase turnover intention. Moral distress is connected to perceived quality of care [80], which itself was shown to be associated with turnover intention [40, 53] in this review. Petrisor et al. [50] pointed out that in the pandemic situation, intensive care nurses could have benefitted from interventions targeting the organizational aspects of workflow since root causes of moral distress should be targeted. Additionally, the opportunity to get consultation by ethics committee in case of moral distress could be considered [51].

### Strengths and limitations

This study shows some strengths and limitations. Through data assessment, analysis, and interpretation, a team of researchers with different professional backgrounds (i.e., nursing, public health, psychology) was involved. We used researcher triangulation to ensure data quality. After our initial search covering this up-to-date topic, an alert was installed in order not to miss any new publications during the analysis and writing process. This way, publications until 31 December 2021 were accounted for to grant the most recent coverage. Furthermore, articles from all countries were included to offer a wide range of perspectives and experiences, e.g., because countries were affected differently by the pandemic. One might argue that this is also a weakness because health care systems might not be comparable across nations, but some relationships were found in several countries (e.g., association of turnover intention with moral distress) and get more emphasis this way.

However, there are also some limitations to report. Our systematic search only included publications in English or German. Therefore, we might have missed peer-reviewed articles from national journals. However, we did not include any articles in German, which might lead to the notion that we covered the majority of relevant publications. Furthermore, we did not accept preprints, although their quality is comparable to peer-reviewed articles [81]. With our strategy to create an alert and to thereby include all relevant articles until 31 December 2021, we tried to account for the most recent peer-reviewed publications.

Due to the heterogeneity of the included articles and the incoherent operationalization of turnover intention, we could not conduct a quantitative meta-analysis. The description of how turnover intention was assessed was incomplete in so many articles that we cannot give any synthesized information on factors specifically associated with organizational or professional turnover.

This study was developed from a thesis by the main researcher (K.T.). To account for the latest publications and to ensure data quality through researcher triangulation, we repeated the whole selection process with two reviewers (K.T. and K.H.). The knowledge of the thesis could have influenced the researchers’ judgement during the selection process. However, we discussed each result thoroughly in case of doubts no matter if it was included in the thesis or not. By the time this study was realised, the main researcher (K.T.) was enrolled as a student but closely supervised by U.T. and K.H., who has experience in conducting systematic reviews [82].

## Conclusions

This systematic literature review identified numerous factors associated with nurses’ turnover intention during the COVID-19 pandemic. On the one hand, organizational factors were found to be associated with nurses’ turnover intention. These work-related issues and psychosocial working conditions could be addressed by the employer, e.g., the form of leadership. Hence, this review delivers starting points for organization-wide (e.g., leadership support training for supervisors) or COVID-19-specific interventions (e.g., special offers for recently deployed nurses). On the other hand, individual factors were shown to be associated with nurses’ turnover intention. These characteristics could help to define high-risk groups that are worth to be taken into account for and maybe also involved in the planning of interventions.

Future research should look more precisely into the relationships of cause and effect around the phenomenon of turnover intention in a crisis situation like a pandemic. Longitudinal studies could help in understanding the complex associations of individual and/or organizational factors with turnover intention. Furthermore, more in-depth qualitative methods and the use of mixed-methods approaches could give more insights into the complex reasons for turnover intention from the nurses’ point of view. In general, different forms of turnover intention, e.g., organizational or professional turnover, should be carefully distinguished and precisely defined and operationalized in future research.

## Supplementary Information

**Additional file 1.** 

## Data Availability

All data generated or analysed during this study are included in this published article [and its supplementary information files].

## Abbreviations

COVID-19: Coronavirus Disease 2019
MERS: Middle East respiratory syndrome
PTSD: Post-traumatic stress disorder
SARS: Severe acute respiratory syndrome
WHO: World Health Organization
