# Supplementary material for: Correlates of turnover intention among nursing staff in the COVID-19 pandemic: a systematic review
Source: BMC Nurs. 2022 Jul 4;21:174. doi: 10.1186/s12912-022-00949-4 (PMC9252069; doi:10.1186/s12912-022-00949-4)
Supplement: Supplementary file 1 — Additional file 1. [file 12912_2022_949_MOESM1_ESM.docx]

# Appendix

Search string for systematic search of CINAHL, PsycINFO, PSYNDEX, PsycArticles, SocINDEX, and MEDLINE via EBSCOhost

((MH "Nursing Staff+") OR nurs* OR *Pflege*) AND (COVID-19* OR SARS-CoV-2* OR coronavirus* OR pandemi*) AND ("intent* to leave" OR "intent* to quit" OR "turnover intention*" OR "retention" OR (MM "Personnel Retention") OR "leaving intention*" OR "consider* attrition" OR "consider* resignation" OR “consider* quit*” OR (Beruf*aufg* AND (*Absicht* OR Inten*)) OR (Beruf*wechsel* AND (*Absicht* OR Inten*)) OR (Stelle*wechsel* AND (*Absicht* OR Inten*)) OR (Beruf*ausst* AND (*Absicht* OR Inten*)) OR (Kündig* AND (*Absicht* OR Inten*)))
